# Upcycling Low-Quality Cotton Fibers into Mulch Gel Films in a Fast Closed Carbon Cycle

**DOI:** 10.3390/gels10040218

**Published:** 2024-03-23

**Authors:** Shaida S. Rumi, Sumedha Liyanage, Zhen Zhang, Noureddine Abidi

**Affiliations:** Department of Plant and Soil Science, Fiber and Biopolymer Research Institute, Texas Tech University, Lubbock, TX 79409, USA; shaida-sultana.rumi@ttu.edu (S.S.R.); sumedha.liyanage@ttu.edu (S.L.)

**Keywords:** cotton fibers, mulch gel films, soil degradation, cellulose, upcycle

## Abstract

Low-quality cotton fibers, often overlooked as low-value materials, constitute a marginalized waste stream in the cotton industry. This study endeavored to repurpose these fibers into mulch gel films, specifically exploring their efficacy in covering moisture-controlled soil beds. Through a meticulously designed series of processing methods, cellulose/glycerol film was successfully fabricated by regenerating cellulose hydrogels in N,N-dimethylacetamide/lithium chloride solutions, followed by plasticization in glycerol/water solutions and hot pressing. The film was then employed to cover soil beds for a duration of up to 252 days, followed by soil burial assessments. Despite expectations of degradation, the film maintained structural integrity throughout the soil covering period but underwent complete biodegradation after 80 days of soil burial, thereby completing a closed carbon cycle. Intriguingly, both tensile strength and modulus exhibited no diminishment but instead increased after soil covering, contrary to expectations given the usual role of degradation. Mechanistic insights revealed that the removal of glycerol contributed to the mechanical enhancement, while microbial activity predominately decomposed the amorphous regions in soil covering and targeted the crystalline portions in soil burial, elucidating the main biodegradation mechanisms. In summary, this study presents, for the first time, the potential of upcycling low-quality cotton fibers into high-value mulch gel films for agricultural practices within a closed carbon cycle.

## 1. Introduction

According to a recent report [1] from the US Department of Agriculture, global cotton consumption is estimated to reach 116.2 million bales by 2024, representing an increase of over 6.6 million bales compared to 2023. This upward trend in consumption is expected to continue, causing the generation of low-quality cotton fibers and a substantial challenge of underutilized waste streams within the cotton industry. Therefore, the question of how to upcycle these low-value materials into value-added applications to address the growing concerns over sustainability and resource optimization has received tremendous attention [2,3]. So far, upcycling low-quality cotton fibers into dye adsorbents [4,5], composite paper [6], energy devices [7], and antibacterial agents [8] have been reported. However, these low-quality cotton fibers are still overlooked due to their perceived lack of economic viability. 

On the other hand, mulching films have been widely used in modern agricultural practices for growing annual and perennial crops such as cotton, corn, vegetables, and fruits [9]. They offer a range of benefits, such as soil temperature and soil moisture regulation, weed control, and enhanced fertilizer efficiency, contributing to enhanced crop productivity and quality, resource efficiency, and environmental sustainability [10,11,12]. According to a recent report (Market, 2016), the worldwide market value of mulching films was approximately USD 5 billion in 2021 and will reach around USD 6.7 billion by the end of 2026. At present, commonly used agricultural mulching films (i.e., polyethylene [13]) are non-biodegradable, generating large amounts of plastic waste that are difficult to degrade thoroughly in soil, leading to environmental concerns and waste management challenges [14,15]. 

As a result, developing biodegradable plastic mulch films is a promising alternative to address these environmental concerns. Biodegradable polymers, including polylactic acid (PLA), polyhydroxyalkanoates (PHA), poly (butylene succinate) (PBS), poly(butylene succinate-co-adipate) (PBSA), and poly(butylene-adipate-co-terephthalate) (PBAT) [16,17,18], have been reported to serve as mulch films helping mitigate environmental concerns [11]. However, there are still some limitations. First, PBS, PBSA, and PBAT are still fossil-fuel-based polymers, prompting the need for fully bio-based bioplastic films, which would be more favorable for certified organic production in the United States [19]. In addition, many biodegradable mulch products degrade relatively slowly, typically within 1–2 years after soil burial, depending on local climatic conditions [12,20,21]. Therefore, the development of fully bio-based mulch films with rapid biodegradation properties is highly desirable.

Cellulose, the most abundant biodegradable natural polymer on earth [22], is a viable option for developing biodegradable agricultural mulch films. This option can offer various advantages: (i) the cellulose content in low-quality cotton fibers has been reported to be 87.2–99.6 wt % [23], providing a high potential for transforming them into mulch films; (ii) cellulose films can be prepared via solvent regeneration and hot pressing techniques according to our previous study [24]; (iii) the soil degradation rate of cellulose is quick [25], and its residence time has been reported to be 31–61 days and 81–495 days in tropic and temperate forests, respectively [26], providing a fast closed carbon cycle. Therefore, it is feasible to develop cellulose films as agricultural mulch films. To the best of our knowledge, this is the first report regarding upcycling low-quality cotton fibers into mulch films. 

In our previous work, the cellulose/glycerol films were prepared using low-quality cotton fiber in multiple steps, such as solvent regeneration of cellulose N,N-dimethylacetamide (DMAc)/lithium chloride (LiCl) solutions, plasticization in glycerol/water solutions, and hot pressing [24]. However, no biodegradation study was performed. As part of our ongoing work, we aim to utilize the cellulose/glycerol films as entirely bio-based and biodegradable agricultural mulches for application in sustainable and organic farming, and to study their biodegradation behavior in soil. Specifically, cellulose/glycerol films were utilized to cover soil beds for an extended duration of up to 252 days, followed by comprehensive soil burial assessments. Various physicochemical properties, such as thermal and tensile properties, and crystalline structures were investigated. The biodegradation mechanism was also elucidated. 

## 2. Results and Discussion

**Figure 1:**
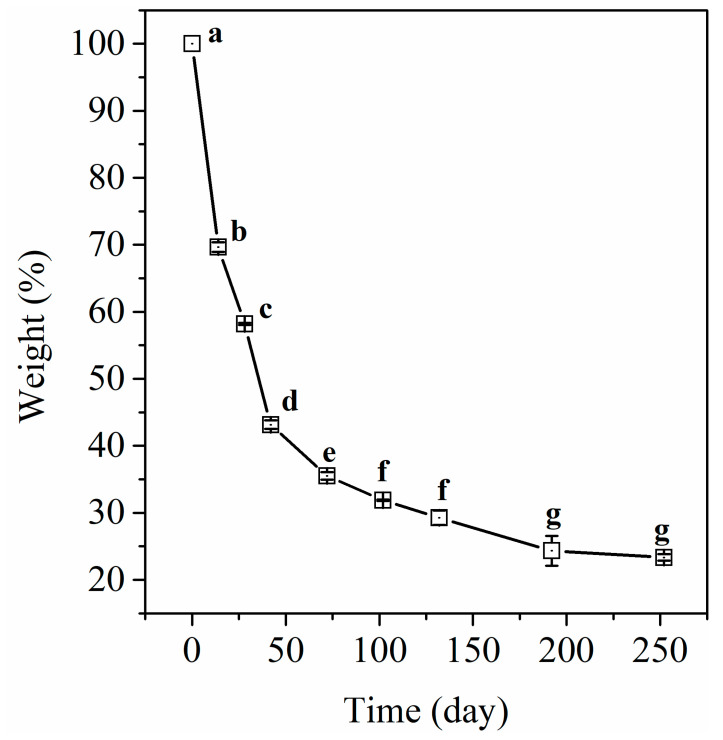
The weight loss of soil cover samples. Values not followed by the same letter significantly differ at α = 0.05.

A gradual weight loss in the soil cover samples was observed over time. As shown in Figure 1, the rapid weight loss that occurred within the initial 42 days could be attributed to two primary factors. First, Lubbock city experiences generally low humidity levels, causing cellulose films to lose a significant quantity of adsorbed water when exposed to the arid environment. Second, the soil beds were regularly watered to maintain a 12 ± 2% soil moisture level. During these watering sessions, glycerol, being highly miscible with water, may have been removed from the films, contributing to weight loss. Glycerol, a sugar alcohol containing three hydroxyl groups, exhibits high solubility in water. This is because it readily adheres to water molecules and establishes effective hydrogen bonds with neighboring molecules, owing to its flexible carbon backbone in diluted solutions [27,28]. In plasticized cellulose films, certain glycerol molecules engage in intermolecular hydrogen bonding with cellulose, while others remain unbound [29]. Consequently, glycerol tends to migrate away from cellulose films, resulting in a loss of mass for the films [27,29]. Beyond day 42, the reduction in weight loss became slower, possibly due to continued glycerol removal and microbial activity of microbes on the cellulose films.

**Figure 2:**
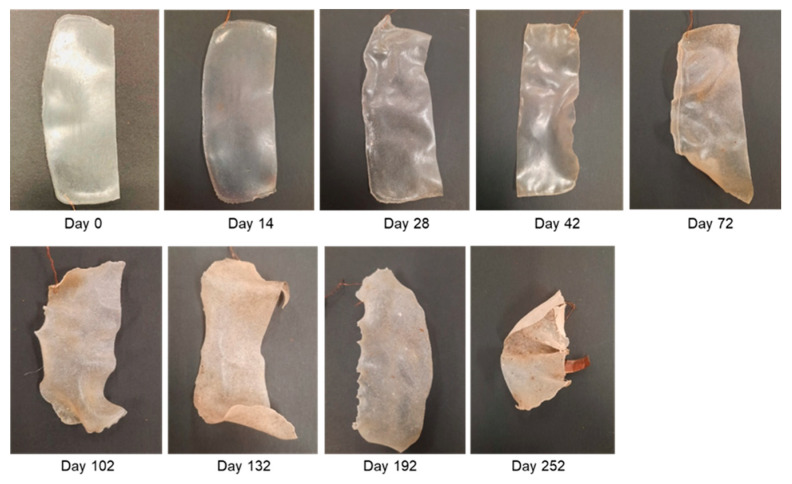
Appearance of soil cover samples as a function of time.

Figure 2 illustrates the gradual transformations in sample appearance during the soil cover experiment. Initially, up to day 42, the cellulose films exhibited slight shrinkage and warp. A transition was observed on day 72, when considerable shrinkage and warp, as well as the appearance of spots and tiny cracks, were observed. On the one hand, the loss of glycerol caused the cellulose films to become progressively stiffer and prone to warping. On the other hand, the cellulose films served as a nutrient source for fungi and microorganisms, fostering their enzymatic activity, which in turn led to the formation of various spots. Interestingly, the material’s structural integrity remained intact even after 252 days of the experiment.

**Figure 3:**
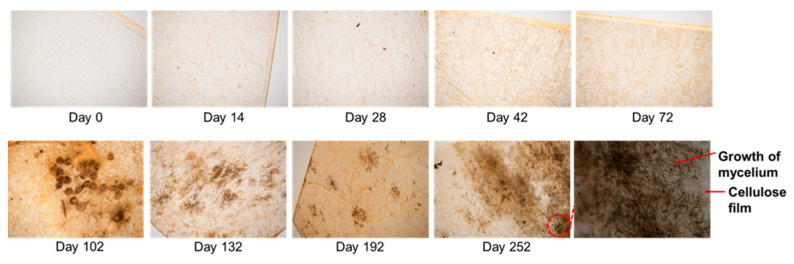
Stereomicroscopic images of soil cover samples as a function of time.

Figure 3 presents stereomicroscopic images of soil cover samples as a function of time. Initially, up to day 72, the formation of textures was visible. A remarkable morphological change occurred on day 102, when the color turned yellow, and some dark spherical objects, likely the reproductive spores of fungi, were observed. These spores serve as the initial stage of fungal reproduction [30], with their germination leading to the formation of hyphae [31]. The rigid walls of these hyphae facilitate their advancement through the substrate, allowing them to grow longitudinally and branch out [31]. Eventually, they develop into a complex network of branching filaments referred to as mycelium (see the enlarged area of day 252). 

**Figure 4:**
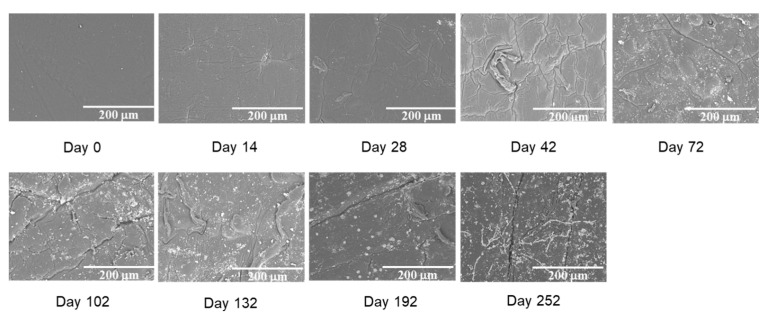
SEM images of soil cover samples as a function of time.

Figure 4 shows the surface morphology of cellulose films throughout the soil cover experiment. Initially, the film exhibited a smooth surface. There were no discernible changes in the surface morphology until day 28. However, from day 72 onwards, subtle indications of surface deterioration became apparent. These included surface irregularities, roughness, and the presence of spherical entities. These small spherical particles could be attributed to either dust particles or microbial aggregations. Notably, the surface of the films retrieved on day 252 exhibited a distinctive radiating network-like structure reminiscent of fungi mycelia, which corresponded well with the previous stereomicroscopy results. 

**Figure 5:**
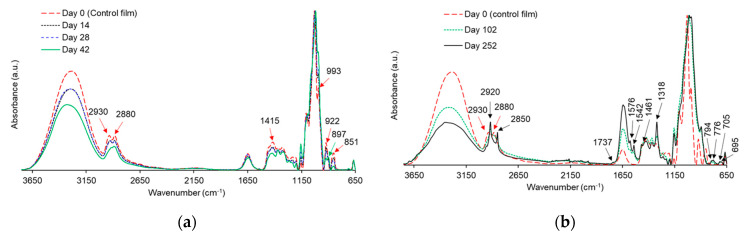
Comparison of FTIR spectra obtained from control cellulose film and soil cover films retrieved at different time intervals (**a**,**b**).

FTIR spectra are displayed in Figure 5a,b. From Figure 5a, control cellulose/glycerol film displayed characterization peaks at 3284 cm^−1^ and 1640 cm^−1^, which were attributed to the OH stretching of cellulose and glycerol and OH bending of adsorbed water, respectively, while other characteristic peaks, at 2930, 2880, 1415, 1115, 993, 922, and 851 cm^−1^, were attributed to the glycerol [24]. With the increasing retrieval time, the glycerol-generated characterization peaks became blunt, and some of them even disappeared. For example, the characteristic peak at 922 cm^−1^ attributed to glycerol was replaced by 897 cm^−1^ for the soil cover sample retrieved from day 42 onwards, which was attributed to the β-linkage of cellulose [24]. These results suggested the removal of glycerol from the films due to the good solubility between glycerol and water [32]. 

When it comes to the later stages of the cover period, some new characteristic peaks were introduced. For example, the new peaks at 2918 and 2850 cm^−1^ (CH_2_ asymmetric and symmetric stretching), 1737 cm^−1^ (carbonyl group), and 1576 and 1542 cm^−1^ (N-H and C-N groups of amide II) were all attributed to the biomolecules present in microorganisms [33,34]. These results corresponded well with the observed mycelium growth in stereomicroscopic analysis (Figure 3) and confirmed the biodegradability of cellulose.

**Figure 6:**
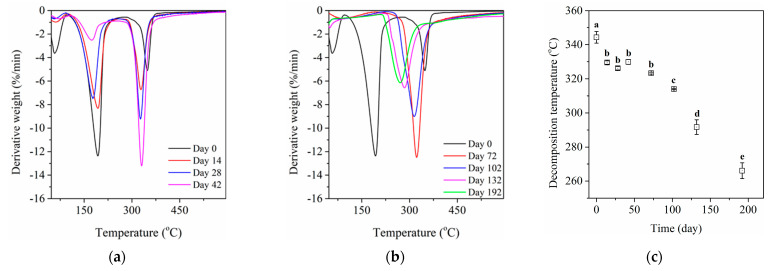
Derivative thermogravimetric curves of the control and soil cover samples (**a**,**b**), and (**c**) cellulose decomposition temperature as a function of the retrieval time. Values not followed by the same letter significantly differ at α = 0.05.

Figure 6a,b displays the derivative thermogravimetric (DTG) curves of the control and soil cover samples. The corresponding TGA curves are listed in Figure S1. Control cellulose/glycerol film displayed two prominent decomposition peaks at around 200 °C and 340 °C, which corresponded to the decomposition of glycerol and cellulose [24,35,36], respectively. Two interesting pieces of information were retrieved. First, with the increasing retrieval time, the DTG peak attributed to the degradation of glycerol gradually diminished (Figure 6a) and eventually disappeared in the soil cover sample retrieved from day 72 onwards. This feature suggested the gradual removal of glycerol, aligning with the aforementioned FTIR results. Second, the DTG peak corresponding to the decomposition of cellulose in soil cover samples exhibited a higher peak intensity and shifted to a lower temperature region, leading to a decrease in the onset decomposition temperature (T_onset_) of cellulose (Figure 6c). These results implied a decrease in the thermal stability of cellulose. The hydrophilic nature of cellulose films provides an environment conducive to microbial colonization [37], which would disturb and disrupt the molecular structure of the polymer and thereby cause a decrease in thermal stability. It should be noted that the thermal stability of films started to decrease significantly from day 132 onwards, which was consistent with the transition (sudden increase) in microbial growth observed in previous stereomicroscopic results (Figure 3).

**Figure 7:**
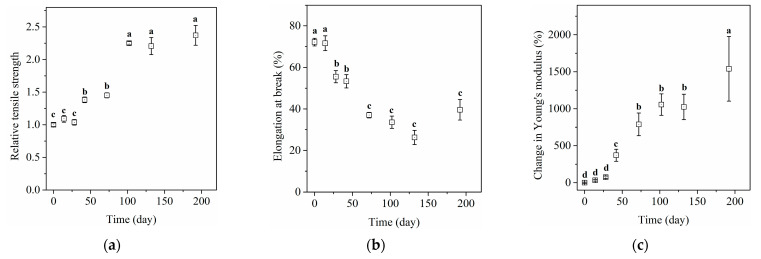
Tensile properties of soil cover cellulose films retrieved at different intervals. (**a**) Relative tensile strength, (**b**) elongation at break, and (**c**) change in Young’s modulus. Values not followed by the same letter significantly differ at α = 0.05.

Tensile properties, including relative tensile strength, elongation at break, and change in Young’s modulus, are summarized in Figure 6a–c. The specific tensile strength and Young’s modulus of different samples can be seen in Figure S2. From Figure 7a,c, both the relative tensile strength and change in Young’s modulus tended to level off until day 28, after which a significant increasing trend was noted. In contrast, the elongation at break showed an overall decreasing trend. It is interesting to note that the trend in tensile strength was in contrast to previous work, where a decreasing trend in tensile strength was observed in polylactic [38] and poly (butylene adipate-co-terephthalate) (PBAT)/starch systems [39]. Such an interesting difference was attributed to the change in glycerol in our current systems. Herein, there was a gradual loss of glycerol during the sprinkler-type irrigation of soil beds, as evidenced by previous FTIR and TGA results. Consequently, the removal of glycerol contributed to the loss of plasticity of cellulose films, making them stiffer and more rigid. 

**Figure 8:**
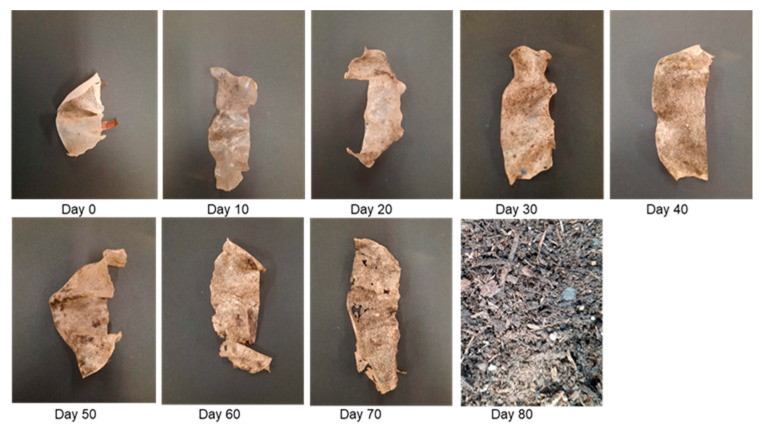
Morphological changes in soil cover samples during their soil burial degradation: visual images.

**Figure 9:**
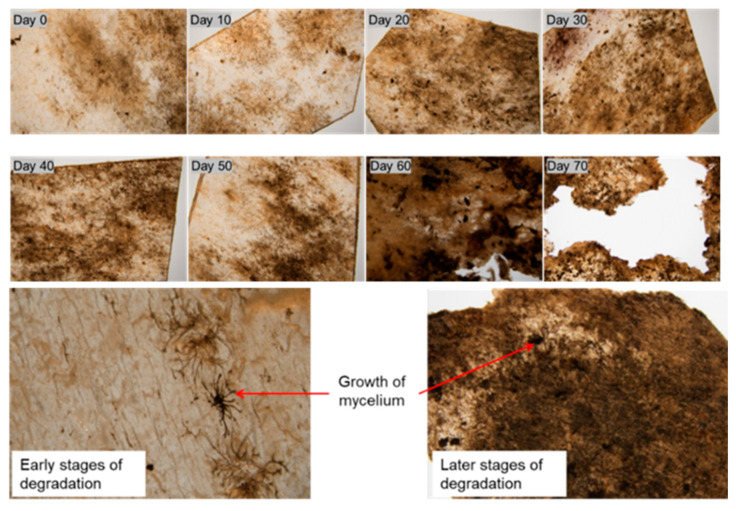
Morphological changes in retrieved soil cover samples: stereomicroscopic images (top) and microbial colonization on cellulose films at early and latter stages of degradation (bottom).

After concluding the soil cover experiment, the remaining films were buried in moist soil beds to investigate their degradation. Figure 8 depicts the appearance of films with increasing burial time. Initially, the films used for the degradation study appeared stiff and folded. With the increasing soil burial time, films interestingly became flexible due to their absorption of soil moisture. Black and white spots (day 10 in Figure 8), later identified as fungal mycelia, were observed on the surface using a stereomicroscope, gradually spreading over the samples (Figure 9—top). Stereomicroscopic images showed increased hyphal branching with retrieval time. It was also observed that cellulose films became progressively darker due to microbial growth on the surface and strong attachment of soil particles (Figure 9—bottom). It was noted that the hyphal network of fungi extended into the internal structure of the cellulose films from the surface, which indicated that the microbes started feeding on cellulose films from the surface. As the mycelia continuously spread, on day 60, visible holes appeared, and the size of the holes became much larger on day 70 (Figure 9—middle). Finally, after 80 days of soil burial, the films lost their structural integrity (Figure 8), suggesting their complete biodegradation. This characteristic is promising for agricultural practices, as it allows producers to harness the benefits of mulch without long-term residue buildup in the soil, contributing to a sustainable carbon cycle. 

**Figure 10:**
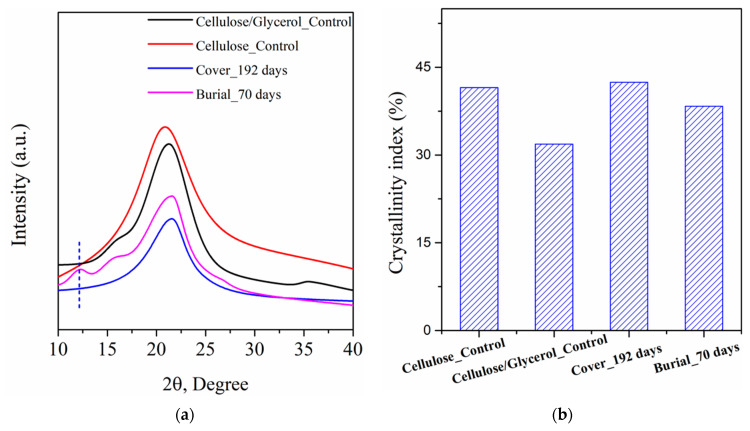
XRD curves (**a**) and crystallinity index (**b**) of different samples.

To gain deeper insights into the degradation mechanisms, XRD patterns of different samples were examined, including cellulose control, cellulose/glycerol control, soil cover at 192 days, and soil burial at 70 days, as depicted in Figure 10a. From Figure 10a, all samples displayed a prominent diffraction peak at around 21°, yet the soil cover and soil burial samples displayed a relatively blunt diffraction peak, indicating possible changes in the crystalline structure [40]. Moreover, a new shoulder peak at ~12.7° was observed in the soil burial at 70 days sample, indicating further alterations in the crystalline structure due to biodegradation. 

The crystallinity indices of different systems are listed in Figure 10b. From Figure 10b, the CI of the cellulose/glycerol control sample was much lower than that of the cellulose control sample, which was attributed to the existence of glycerol [24]. When it comes to degraded samples, it was interesting to note that the CI initially increased for the soil cover at 192 days, followed by a subsequent decrease in CI for soil burial at 70 days. Based on these results, the degradation mechanism was proposed. During the soil cover stage, the removal of glycerol from the film would contribute to the higher CI. In addition, the amorphous region was more susceptible to microbial decomposition than the crystalline regions, thus increasing the CI [41]. Conversely, during the soil burial period, microbial activity may have initially decomposed the amorphous region, subsequently targeting the crystalline portion, leading to a reduction in crystallinity and, ultimately, the loss of the structural integrity of the samples. 

## 3. Conclusions

In summary, our current study highlighted the potential of upcycling low-quality cotton fibers into high-value mulch gel films for sustainable agricultural practices. Via a series of efficient processing methods, including wet chemistry and hot pressing, cellulose/glycerol film was successfully fabricated, which was then applied to moisture-controlled soil beds. The unexpected maintenance of structural integrity throughout the soil covering phase (252 days), followed by complete biodegradation after soil burial (80 days), underscores the closed carbon cycle associated with this agricultural application. Moreover, the unexpected continuous enhancement in tensile strength and modulus observed during the soil cover phase was attributed to the gradual loss of glycerol. The XRD results indicated that the microbial activity initially affected the amorphous region during the soil covering phase and finally targeted the crystalline region. Overall, this study represents a significant advancement in closing the carbon cycle by repurposing waste materials into valuable resources, thereby promoting sustainable agricultural practices.

### Further Directions

The slow degradation of biodegradable mulches has limited their widespread use in vegetable production. However, this study suggests the potential for developing mulches that offer agricultural benefits without the risk of long-term residue buildup in the soil. The faster biodegradability of entirely bio-based films may appeal to an increasing number of producers seeking ways to reduce the time, labor, and environmental impact associated with removing and disposing of petro-plastic mulch. Nevertheless, further research is needed to optimize the physicochemical characteristics of the films, and field trials should be conducted to assess their suitability for open-field application. Additionally, it would be prudent to analyze the films for residual lithium salt content. Studies have shown that the toxicity of LiCl can adversely affect plant growth parameters [42]. Although certain plant species may absorb modest concentrations of lithium salts, which can promote plant growth, high concentrations of Li in soils are detrimental to plants [43]. It inhibits physiological characteristics and limits plant biomass production [44]. Lastly, it is essential to quantitatively characterize the emission of carbon dioxide resulting from the biodegradation of cellulose films. In the future, optimizing the rate of the carbon cycle in a customizable manner may become a significant area of interest.

## 4. Materials and Methods

### 4.1. Materials

Extra pure DMAc (99%, A0403006) and anhydrous LiCl (99%, A0386841) were obtained from Acros OrganicsTM (Fair Lawn, NJ, USA). Glycerol (202397, certified ACS grade) was purchased from Fisher ScientificTM (Fair Lawn, NJ, USA). Low-quality cotton (micronaire = 2.4) was collected locally (Lubbock, TX, USA). Organic potting soil (All-Natural Premium Outdoor Potting Mix, Kellogg Garden Products Corporate, Carson, CA, USA) was purchased from Home Depot. 

### 4.2. Methods

#### 4.2.1. Film Preparation

**Scheme 1:**
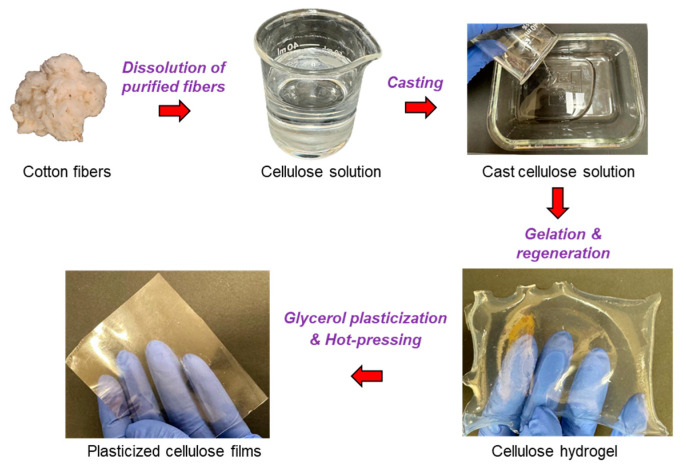
Schematic representation of the preparation process of cellulose/glycerol film.

Cellulose/glycerol films were prepared (Scheme 1) following the procedures reported in our previous work [24]. Briefly, the DMAc/LiCl solvent system was used to dissolve scoured and bleached (purified) cotton fibers. Purified cotton fibers, dried in an oven at 105 °C for 24 h, were added to a hot DMAc solvent at 80 °C and stirred for 30 min. Subsequently, oven-dried LiCl (8% *w*:*v*) was added, and the solution was stirred for an additional 3 h at the same temperature. The temperature was then reduced to 50 °C, and the dissolution process continued overnight, totaling 24 h on the hotplate. The solution was later transferred to an oven set at 105 °C. After 12 h, it was withdrawn from the oven and allowed to cool to room temperature. The resultant solution was cast in glass molds; the cellulose hydrogel films were regenerated in DI water, further plasticized using 30% glycerol, and hot pressed. The cellulose/glycerol films were placed in a conditioning room for at least 48 h (relative humidity of 30 ± 2% and temperature of 21 °C) before further characterization. The dimensions of each cellulose film were recorded, and then each film was labeled with copper tape bearing a unique identification number.

#### 4.2.2. Soil Cover Experimental Setup

**Scheme 2:**
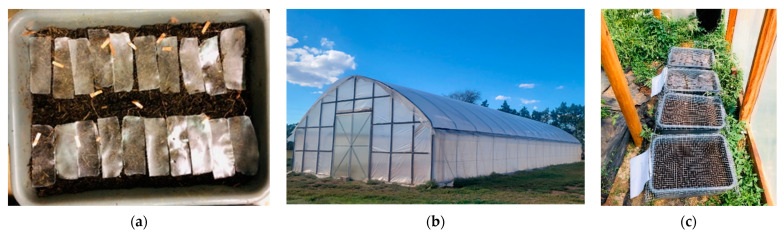
Cellulose films exposed to a moisture-controlled environment: (**a**) cellulose films placed on the soil bed of a preconditioned soil tray (12 ± 2% moisture), (**b**) high tunnel, and (**c**) sample trays placed inside the high tunnel.

The pre-moistened organic potting soil was arranged into 10 cm deep trays (Scheme 2a) and brought to a moisture level of 12 ± 2% through controlled watering and seven days of conditioning. To optimize microbial activity, it is advisable to maintain moisture levels between 40% and 50% of the soil’s maximum water-holding capacity [45]. Considering the soil in this experiment had a 25% water-holding capacity, a moisture content range of 10% to 12.5% would be suitable. Additionally, for optimal seed germination and seedling growth, a soil moisture content of around 12% is recommended [46]. Therefore, a soil moisture level of 12 ± 2% was selected for our investigation. Soil trays were then transferred to a high tunnel (Scheme 2b). Cellulose/glycerol films were then placed on the soil beds (Scheme 2a) for 252 days while maintaining soil moisture at 12 ± 2% throughout the experiment using a digital soil moisture meter (DSMM500, General Tools & Instruments, Secaucus, NJ, USA) and periodic watering. This experiment was carried out with two replications, and during each retrieval, two cellulose films were taken out of each soil bed. 

At the end of the soil cover experiment, the remaining cellulose films were buried in moist soil beds to investigate their degradation. The same soil trays used for the cover experiment were employed for this subsequent experiment. The films were buried in moist soil beds (5 cm below the soil surface). The films were interred in moist soil beds and then lightly hand-compacted to ensure good contact between the samples and the soil layers. Throughout the experiment, a soil moisture level of 12 ± 2% was maintained, and at ten-day intervals, two cellulose films were retrieved from each tray for analysis.

### 4.3. Characterizations 

After retrieving the cellulose films, they were cleaned with a brush to remove as many attached soil particles as possible. Subsequently, visual images of the samples were captured. The cleaned cellulose films were conditioned at 65 ± 2% relative humidity and 21 ± 1 °C temperature for 48 h before characterization. 

#### 4.3.1. Weight Change during the Soil Cover Period

The samples were conditioned at ambient room temperature for at least 48 h, and their weights were recorded immediately before the experiment and after each retrieval. The percent weight changes during the cover period were calculated using the following equation:

$$Weight\;change\;(\%)=\frac{Initial\;weight-Weight\;after\;retrieval}{Initial\;weight}\times 100$$

#### 4.3.2. Surface Morphology

The morphological changes in the cellulose films were characterized using a stereomicroscope (SMZ800N, Nikon Corporation, Tokyo, Japan) and a scanning electron microscope (SEM, TM-1000, Hitachi, Japan). Cellulose films were placed on the sample stage and visualized with a 1× objective lens of the stereomicroscope. For SEM tests, samples were mounted on the sample stage using carbon tape (Ted Pella Inc., Redding, CA, USA) and visualized at 500× magnifications with an accelerating voltage of 15 kV. 

#### 4.3.3. Thermogravimetric Analysis (TGA)

The thermal properties of the cellulose films were analyzed using a Pyris 1 thermogravimetric analyzer (PerkinElmer, Shelton, CT, USA) equipped with an autosampler. Small pieces of cellulose films were placed in the crucibles of the autosampler, and thermograms were recorded between 37 and 600 °C with a heating rate of 10 °C/min under a constant flow of nitrogen (20 mL/min). Pyris data analysis software (Version 13.3.2, PerkinElmer, Shelton, CT, USA) was used to calculate the derivative thermograms (DTG) and the decomposition temperatures of the samples.

#### 4.3.4. Fourier Transform Infrared (FTIR) Tests

FTIR spectra of the cellulose films were recorded using an FTIR spectrometer (Spectrum 400, PerkinElmer, Boston, MA, USA) equipped with a ZnSe diamond crystal and a pressure arm. In each test, spectra were recorded from 4000 to 650 cm^−1^ (4 cm^−1^ spectral resolution and 32 co-added scans). Baseline correction and normalization were performed using Spectrum software (Version 10.5.3, PerkinElmer, Boston, MA, USA). 

#### 4.3.5. Tensile Testing

Tensile testing of the cellulose films was performed as per ASTM 638-14 using the Multi-Test 2.5-dV(u) Test System (Mecmesin, West Sussex, UK). The griping distance and the speed of the instrument were set to 65 mm and 50 ± 10 mm/min, respectively. A tensile die (ASTM D638-Type IV) and a manual clicker press (Qualitest, Bridgewater, NJ, USA) were utilized to obtain the standard dimensions of each sample. 

#### 4.3.6. X-Ray Diffraction (XRD) Analysis

XRD patterns were recorded in an XRD diffractometer (Smartlab HD2711n, Rigaku Corporation, Tokyo, Japan) coupled with Cu Kα radiation (λ = 0.154 nm; 40 kV and 44 mA). Each film sample was scanned from 10 to 30° at a scanning rate of 1°/min. The Segal method [47,48] was utilized to calculate the crystallinity index (CI).

#### 4.3.7. Statistical Analysis

One-way analysis of variance (ANOVA) was conducted with the retrieval time of cellulose films as a factor to determine the statistical difference at a 95% confidence interval (*p* ≤ 0.05) using TIBCO STATISTICA (Version 13.3; March 2021; Palo Alto, CA, USA). In addition, the Newman–Keuls method (post hoc) was used to ascertain the differences between different retrieval times.

## Data Availability

Data will be made available upon reasonable request.

## Supplementary Materials

The following supporting information can be downloaded at https://www.mdpi.com/article/10.3390/gels10040218/s1, Figure S1: TGA thermograms of (a) the control and soil cover cellulose films (day 0–day 72) and (b) the soil cover cellulose films (day 102–day 192); Figure S2: Tensile properties of cellulose films retrieved from the soil cover experiment: (a) tensile strength and (b) Young’s modulus.
