# Supplementary material for: Upcycling Low-Quality Cotton Fibers into Mulch Gel Films in a Fast Closed Carbon Cycle
Source: Gels. 2024 Mar 23;10(4):218. doi: 10.3390/gels10040218 (PMC11049410; doi:10.3390/gels10040218)
Supplement: Supplementary file 1 [file gels-10-00218-s001.zip › gels-2926725-supplementary.pdf]

Supporting Information:

Upcycling Low-Quality Cotton Fibers into Mulch Materials in A Fast Closed Carbon Cycle

Shaida S. Rumi, Sumedha Liyanage, Zhen Zhang*, Noureddine Abidi*

Fiber and Biopolymer Research Institute, Department of Plant and Soil Science, Texas Tech University, Lubbock, Texas, USA 79409

*Corresponding authors: Zhen Zhang (Email: zha03518@ttu.edu); Noureddine Abidi (E-mail: noureddine.abidi@ttu.edu)

a.

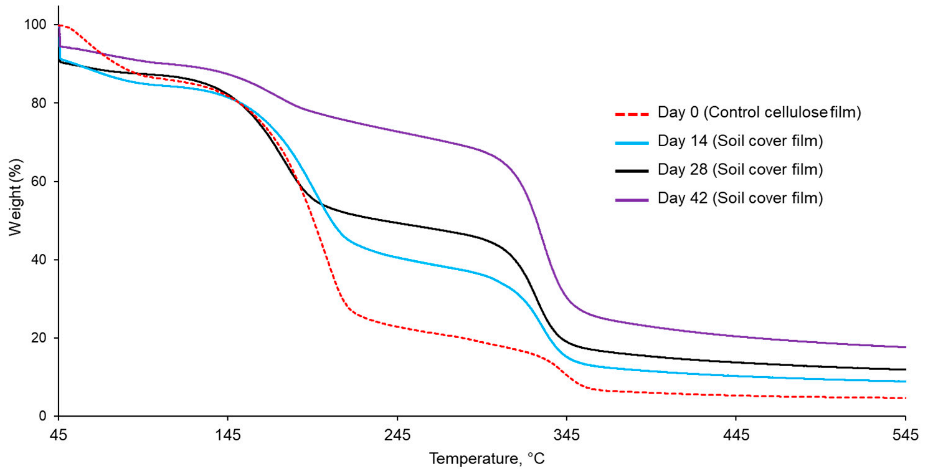

b.

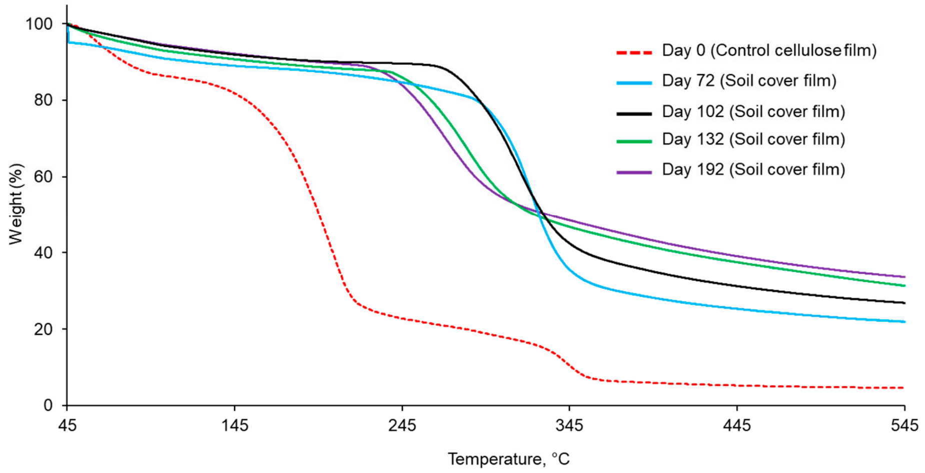

Figure S1 TGA thermograms of the control and soil cover films from day 0-day 42 days (a) and (b) day 72- day 192.

a.

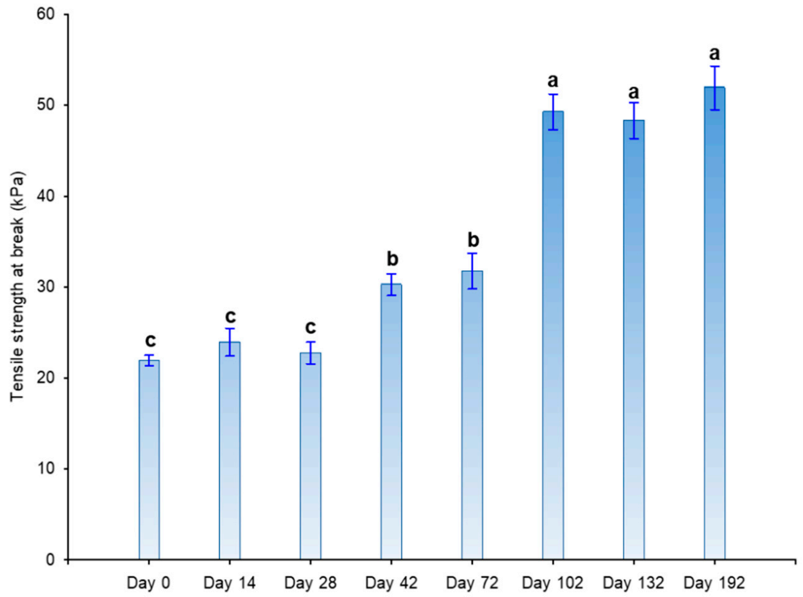

b.

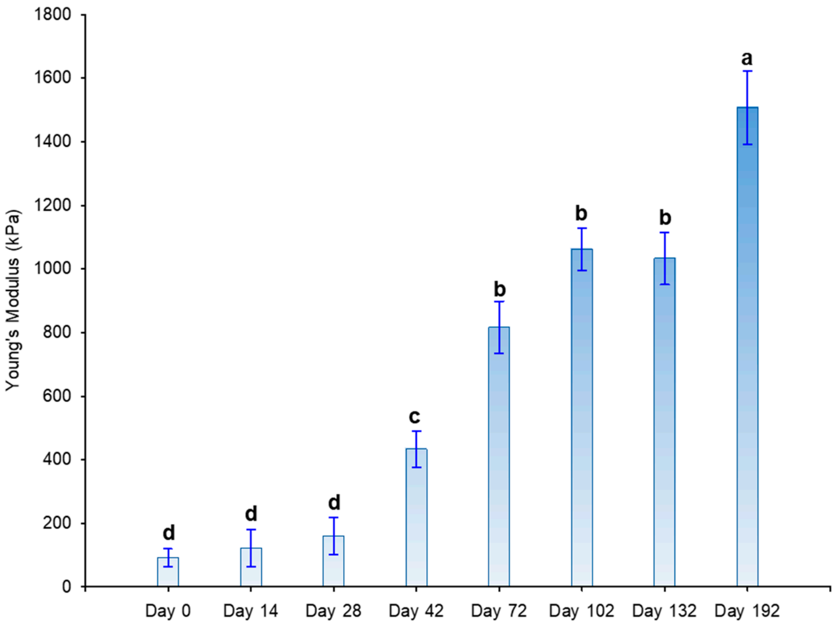

Figure S2 Tensile properties of cellulose films retrieved from soil cover experiment: (a) tensile strength; (b) Young's Modulus. Values not followed by the same letter significantly differ at $\alpha = 0.05$.
